# Genome-Wide Identification and Characterization of the Biosynthesis of the Polyamine Gene Family in *Citrus unshiu*

**DOI:** 10.3390/genes14081527

**Published:** 2023-07-26

**Authors:** Saleha Sadiq, Mujahid Hussain, Shahid Iqbal, Muhammad Shafiq, Rashad Mukhtar Balal, Mahmoud F. Seleiman, John Chater, Muhammad Adnan Shahid

**Affiliations:** 1 Department of Horticulture, Faculty of Agricultural Sciences, University of the Punjab, Lahore 54590, Pakistan; 2 Horticultural Science Department, North Florida Research and Education Center, University of Florida/IFAS, Quincy, FL 32351, USA; 3 Department of Horticulture, College of Agriculture, University of Sargodha, Sargodha 40100, Pakistan; 4 Department of Plant Production, College of Food and Agriculture Sciences, King Saud University, Riyadh 11451, Saudi Arabia; 5 Horticultural Science Department, Citrus Research and Education Center, Lake Alfred, FL 33850, USA

**Keywords:** abiotic stress, *Citrus unshiu*, polyamines

## Abstract

Polyamines (PAs) contribute to diverse plant processes, environmental interaction, and stress responses. In citrus, the mechanism underlying the biosynthesis of polyamines is poorly understood. The present study aims to identify the biosynthesis of PA gene family members in satsuma mandarin (*Citrus unshiu*) and investigate their response against various stresses. The identified biosynthesis of PA genes in *C. unshiu* showed clustering in six groups, i.e., *SPMS*, *SPDS*, *ACL5*, *ADC*, *ODC*, and *SAMDC*. Syntenic analysis revealed that segmental duplication was prevalent among the biosynthesis of PA genes compared to tandem duplication. Thus, it might be the main reason for diversity in the gene family in *C. unshiu*. Almost all biosynthesis of PA gene family members in *C. unshiu* showed syntenic blocks in the genome of *Arabidopsis*, *Citrus sinensis*, *Poncirus trifoliata*, and *Citrus reticulata*. Analysis of *Cis*-regulatory elements (CREs) indicated the occurrence of hormones, light, defense, and environmental stress responses as well as the development and other plant mechanisms-related elements in the upstream sequence of the biosynthesis of PA genes. Expression profiling revealed that the biosynthesis of PA gene expression modulates in different organs during various developmental stages and stress in *C. unshiu*. This information will provide a deep understanding of genomic information and its expression in multiple tissues to better understand its potential application in functional genomics.

## 1. Introduction

Polyamines (PA) are low molecular weight, small linear/aliphatic, or sometimes branched polycationic nitrogen-containing compounds, including unsaturated hydrocarbons with two or more primary amino groups [1,2,3,4]. At normal cell pH, the structure of PA is characterized by cations and contains methylene. According to reports, these methylene groups participate in hydrophobic interactions and significantly affect the activity of PA [5]. In addition, as PAs are cationic, they can react with negatively charged macromolecules such as acidic phospholipids, nucleic acids (DNA and RNA), and various proteins [6], helping them to stabilize their structure, especially during stress. Putrescine (diamine), spermidine (triamine), and spermine (tetramine) are the most common PAs in higher plants [7,8]. However, numerous different PAs, for instance, the triamines sym-norspermidine (C6H17N3) and symhomospermidine (C8H21N3); the diamines 1,3-diamino propane (C3H10N2) and cadaverine (C5H14N2); the tetra-amines symnorspermine (C9H24N4) and thermospermine (C10H26N4); and relatively long or branched polyamines are relatively uncommon [8].

*C. unshiu* is a cold-hardy species, and the fruit grows sweeter when cultivated in cold climates due to the lower temperatures. A fully matured satsuma tree can withstand temperatures as low as −9 °C (15 °F) to −11 °C (12 °F) for a few hours [9]. Satsumas could be grafted onto other citrus rootstocks, such as trifoliate orange, or germinated from seeds, which takes up to eight years or more [10]. The genus Citrus of the family Rutaceae is among the most cultivated fruit crops on the planet [11]. Citrus spp. could thrive in latitudes ranging from 35° to 40° degrees north and south [11,12]. Hence, they are known to be tropical or subtropical [13].

Previously, organic acids, phytohormones, amino acids, fatty acids, secondary metabolites, and polyamines were identified and associated with physiological functions in citrus plants. PAs are involved in plant development, growth, and several physiological mechanisms in citrus, such as embryogenesis [14,15], root morphology, and development [16,17]; vegetative growth [18,19,20]; flowering [21,22]; growth parameters [18,23]; and photosynthetic pigments [7,24]. Unfortunately, the underlying molecular mechanisms of these roles in *C. unshiu* are still unclear.

In citrus, PAs regulate multiple plant growth and developmental processes. PAs play significant multiregulatory functions at several citrus plant developmental mechanisms and growth stages. Even though our understanding of these functions is yet confined, several functions are still to be investigated. In the available reports, there is still no comprehensive research on the genome-wide identification of the biosynthesis of polyamine gene families in *C. unshiu*. For this study, we conducted a genome-wide analysis of the biosynthesis of PA family genes and analyzed expression profiles. Our research provides a basis for subsequent functional genomics studies of the biosynthesis of the PA gene and its potential role in regulation during various developmental stages and in response to different stresses in *C. unshiu* at the molecular level.

## 2. Materials and Methods

### 2.1. Plant Material, Growth Condition and Transcriptome Analysis

Eight-year-old plants of satsuma mandarin (*C. unshiu* Marc.) cultivar “Yamashitabeni wase” grafted onto the trifoliate orange (*P. trifoliate* L.) rootstocks were exposed to peel roughing disorder of satsuma mandarin. The experiment was performed at the greenhouse at the University of the Punjab, Pakistan in April 2023. The grafted plants were developed in a greenhouse with a photoperiod of 16/8 h (day/night) at 25 C and at a constant relative humidity of 70% for two months. The data was collected after specific time intervals of 30 days, 80 days, and 170 days. A total of 6 samples were taken from both affected and control groups, with three from each group, and kept at −80 °C until further research.

To analyze the external stimuli-specific expression profile of CuSPMS, CuSPDS, CuACL5, CuADC, CuODC, and CuSAMDC in various organs, we obtained previously generated RNA-seq data of plants under several biotic, abiotic, and physiological stresses from National Center for Biotechnology Information- Gene Expression Omnibus (NCBI-GEO) (https://www.ncbi.nlm.nih.gov/geo/ (accessed on 16 April 2023)) [25,26]. These stresses include peel roughing disorder of satsuma mandarin, seedling etiolation, and light drought. Heatmap Illustrator in TBtools (v 1.09) with parameters (cluster: complete; dist method: euclidean; scale: normalized) displayed expression patterns with hierarchical clustering [27].

### 2.2. Database Search, Sequence Retrieval, and Physiochemical Properties

The amino acid sequence of *SPMS*, *SPDS*, *ACL5*, *ADC*, *ODC*, and *SAMDC* was retrieved from *C. unshiu* (v1.0) Mikan genome database (MiGD) (https://mikan.dna.affrc.go.jp/index.html (accessed on 16 April 2023)) [28]. Amino acids (AA) sequence of SPMS domain (PF01564), SPDS domain (PF17284), *ACL5* domain (PTHR43317), *ADC* domain (PF02784), and *SAMDC* domain (PF01536) was retrieved from *Arabidopsis thaliana*, while ODC domain (PF00278) was retrieved from *C. sinensis*, as *A. thaliana* lack ODC genes, using Pfam database (https://pfam.xfam.org/ (accessed on 16 April 2023)). These domains’ peptide sequences were used to identify *SPMS*, *SPDS*, *ACL5*, *ADC*, *ODC*, and *SAMDC* protein-encoding genes in the *C. unshiu*.

Furthermore, the protein sequences of the reported biosynthesis of *Arabidopsis* PA family genes were retrieved from TAIR at Phytozome v13 (https://phytozome-next.jgi.doe.gov/info/Athaliana_TAIR10 (accessed on 16 April 2023)), which consisted of two AtADC genes (AT2G16500 and AT4G34710), two *AtSPDS* genes (AT1G23820 and AT1G70310), single *AtSPMS* gene (AT5G53120), and single AtACL5 gene (AT5G19530). In contrast, CsODC (CISIN_1g015304mg) was retrieved from NCBI (https://www.ncbi.nlm.nih.gov/protein/ (accessed on 17 April 2023)). These sequences were then used for BLAST-P (Protein- basic local alignment search tool) search in *C. unshiu*, and SequnceServer 1.0.13 (https://mikan.dna.affrc.go.jp/blast/ (accessed on 17 April 2023)) was used to reconfirm the retrieved *SPMS*, *SPDS*, *ACL5*, *ADC*, *ODC*, and *SAMDC* proteins in *C. unshiu*. The retrieved amino acid sequences were subjected to NCBI CDD (Conserved Domain Database) (https://www.ncbi.nlm.nih.gov/Structure/cdd/wrpsb.cgi (accessed on 17 April 2023)) with the default parameters [29].

The protein length (amino acid residues), theoretical pI, and molecular weight of *CuSPMS*, *CuSPDS*, *CuACL5*, *CuADC*, *CuODC*, and *CuSAMDC* proteins were predicted using ProtParam tool (https://web.expasy.org/protparam/ (accessed on 20 April 2023)) with software built-in default parameters [30]. The information about the gene IDs, location, gene sequence, and protein were retrieved from Mikan genome database (MiGD). Subcellular localization of *CuSPMS*, *CuSPDS*, *CuACL5*, *CuADC*, *CuODC*, and *CuSAMDC* was predicted using the online tool WoLF PSORT with default built-in parameters (https://wolfpsort.hgc.jp/ (accessed on 20 April 2023)) [31].

### 2.3. Multiple Sequence Alignment, Phylogenetic Analysis, and the Biosynthesis of PA Gene Orthologs in Arabidopsis

The amino acid sequences of *SPMS*, *SPDS*, *ACL5*, *ADC*, *ODC*, and *SAMDC* proteins of three species, *C. unshiu*, *A. thaliana*, and *Citrus sinensis*, were aligned using muscle alignment tool and a phylogenetic tree was created through MEGA X v2.0 [32] program with the neighbor-joining (NJ) method and bootstrap was set at 1000 replications with partial deletion.

Protein homologs of *SPMS*, *SPDS*, *ACL5*, *ADC*, *ODC*, and *SAMDC* genes of *C. unshiu* were identified in *A. thaliana* through protein–protein BLAST (blastp) on NCBI. The analysis was conducted using protein sequences of *SPMS*, *SPDS*, *ACL5*, *ADC*, *ODC*, and *SAMDC* genes against the whole genome of *A. thaliana* (taxonomy id: 3702) with all parameters set as default.

### 2.4. Conserved Motifs, Cis-Element, and Gene Structural Analysis

Multiple EM for Motif Elicitation (MEME) program (https://meme-suite.org/meme/ (accessed on 21 April 2023)) was used to analyze motifs in the retrieved *CuSPMS*, *CuSPDS*, *CuACL5*, *CuADC*, *CuODC*, and *CuSAMDC* protein sequences with the maximum number of motifs set as 20; the width of motif was set as default values along with other factors [33]. A visual representation of the motifs was created using the Gene Structure View (Advanced) software in TBtools with default parameters [34]. A sequence of 1000 bps upstream was retrieved from the initiation codon to analyze the promoter region. PlantCare database (https://bioinformatics.psb.ugent.be/webtools/plantcare/html/ (accessed on 21 April 2023)) was then used to predict the *cis*regulatory elements in these sequences [35].

To find out the arrangement of intron/exon of *SPMS*, *SPDS*, *ACL5*, *ADC*, *ODC*, and *SAMDC* genes, the genomic sequences and genomic feature file of identified genes in *C. unshiu* were retrieved and used to draw the gene structure. The motifs were visually represented using the gene structure view (advanced) software in TBtools with default parameters [27].

### 2.5. Scaffold Location, Synteny Analysis, and Gene Duplication Event

The information regarding scaffold length and location of genes was extracted from the *C. unshiu* genome database, and gene location was visualized through Tbtools. Gene pairs of *C. unshiu* such as *SPMS*, *SPDS*, *ACL5*, *ADC*, *ODC*, and *SAMDC* genes of *C. unshui* were created through Tbtools, and synteny analysis was conducted using the Advanced Circos program in Tbtools with default parameters [36]. The genome and gff3 files of *C. unshiu* were retrieved from Mikan genome database (MiGD) and *C. sinensis* from citrus genome database (https://www.citrusgenomedb.org/ (accessed on 25 April 2023)). In contrast, those of *A. thaliana*, and *Solanum tuberosum* were retrieved from Phytozome database (https://phytozome-next.jgi.doe.gov/ (accessed on 25 April 2023)) to display the syntenic blocks of *SPMS*, *SPDS*, *ACL5*, *ADC*, *ODC*, and *SAMDC* genes of *C. unshiu* in the genomes of *C. unshiu*, *A. thaliana*, *S. tuberosum*, and *C. sinensis*. The syntenic maps were constructed using the One Step MCScanX and Dual Systeny Plot in TBtools with default parameters.

The number of nonsynonymous (Ka) and synonymous (Ks) substitution rates and Ka/Ks ratios were calculated using the simple Ka/Ks calculator through TBTools software [27]. The parameters were set as described inside the software package manual. The Ka/Ks ratios were used to predict the rates of molecular evolution of each paralogous gene pair of *CuSPMS*, *CuSPDS*, *CuACL5*, *CuADC*, *CuODC*, and *CuSAMDC* genes [37]. Generally, a Ka/Ks ratio greater than one indicates positive selection and a ratio of close to 1 indicates neutral selection. In contrast, a ratio of less than 1 indicates the probability of purifying selection, which leads to limited functional divergence of the duplicated genes [38].

### 2.6. Effect of Peel Roughing Disease Stress on C. unshiu PA Biosynthestic Genes

The experiment for transcriptome profiling of *C. unshiu* plants during a physiological disorder was designed to expose eight-year-old plants of “Yamashitabeni wase” cultivar of satsuma mandarin (*C. unshiu* Marc.) grafted onto the trifoliate orange (*P. trifoliate* L.) rootstocks were exposed to peel roughing disorder of satsuma mandarin. The data was collected after specific time intervals of 30 days, 80 days, and 170 days. A total of 6 samples were taken from both affected and control groups, i.e., three from each group [39]. Transcriptomic data was generated through high throughput sequencing (GSE100512) to understand the variation in gene expression of *CuSPMS*, *CuSPDS*, *CuACL5*, *CuADC*, *CuODC*, and *CuSAMDC* in response to the stress, and a graph and heatmap were created to display the pattern.

### 2.7. Citrus unshiu Plant under Seedling Etiolation

To understand the signaling pathways and gene regulation associated with etiolation in citrus, two citrus hybrid cultivars Shiranuhi (*C. unshiu* x *C. sinensis*) x *C. reticulata*) and Huangguogan (*C. reticulata* x *C. sinensis*) were studied for this purpose. The seedling of the two hybrids was grown under light-deprived conditions. The variance in global gene expression between etiolated seedlings and the control group was carried out through high-throughput Illumina sequencing (GSE90935).

### 2.8. Light Drought Effect on C. unshiu PA Biosynthetic Genes during Induction of Flowering Branches

To understand the relationship between the development of flowering branches during drought conditions and the sequence of molecular changes and gene expression modulation responsible for producing flowering in *C. unshiu*, plants were exposed to light drought conditions for 75 days. Then, gene expression variance (GSE202927) was studied between light drought (LD) by comparing them with a conventionally watered group (CK).

## 3. Results

### 3.1. Identification and Characterization of the Biosynthesis of the PA Gene Family in C. unshiu

To identify the *SPMS*, *SPDS*, *ACL5*, *ADC*, *ODC*, and *SAMDC* genes in *C. unshiu*, the protein sequence of *SPMS*, *SPDS*, *ACL5*, *ADC*, *ODC* domains from *C. unshiu* retrieved from Pfam were BLAST against the whole genome of *C. unshiu* (*C. unshiu* v1.0). An initial investigation identified two *SPMS*, four *SPDS*, three *ACL5*, three *ADC*, three *ODC*, and five *SAMDC* proteins. The proteins encoded by the same gene isoforms and proteins containing the truncated domains were excluded from the analysis. Finally, a total of single *SPMS*, two *SPDS*, two *ACL5*, single *ADC*, two *ODC*, and five *SAMDC* nonredundant PA biosynthetic genes were identified and used for further analysis.

*CuSPMS*, *CuSPDS*, *CuACL5*, *CuADC*, *CuODC*, and *CuSAMDC* genes encode proteins ranging from 196 to 681 amino acids in length, having molecular weights that range from 22151.38 to 76854.2 kD, making *CuACL5-1* and *CuADC* the largest proteins. The isoelectric points of *CuSPMS*, *CuSPDS*, *CuACL5*, *CuADC*, *CuODC*, and *CuSAMDC* proteins ranged from 4.96 to 7.2 (Table 1).

### 3.2. Comparative Phylogenetic and Orthologs Analysis of PA Biosynthetic Genes

The phylogenetic analysis of *SPMS*, *SPDS*, *ACL5*, *ADC*, *ODC*, and *SAMDC* proteins of *C. unshiu* genes was conducted and compared with *A. thaliana* and *C. sinensis* genes. To investigate the evolutionary relationships between *CuSPMS*, *CuSPDS*, *CuACL5*, *CuADC*, *CuODC*, and *CuSAMDC; CsSPMS*, *CsSPDS*, *CsACL5*, *CsADC*, *CsODC*, and *CsSAMDC*: and *AtSPMS*, *AtSPDS*, *AtACL5*, *AtADC*, *AtODC*, and *AtSAMDC*, a phylogenetic tree was constructed using the neighbor-joining (NJ) method through MEGA X v10.2.4 by aligning full-length protein sequences.

**Figure 1:**
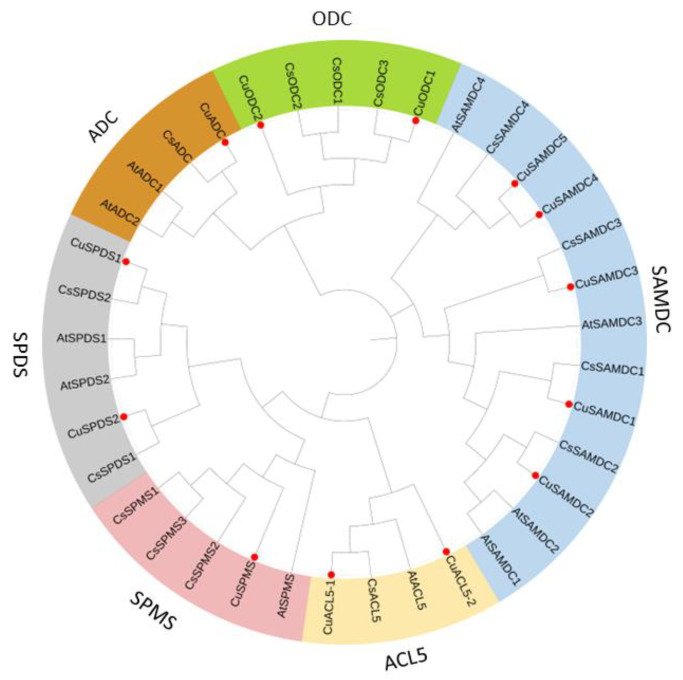
Phylogenetic relationships of *SPMS*, *SPDS*, *ACL5*, *ADC*, *ODC*, and *SAMDC* genes in *C. unshiu* with respective genes belonging to *A. thaliana*, and *C. sinensis*. An online tool, iTol v6 (Interactive Tree of Life), constructed a phylogenetic tree. Red dots highlight the *CuSPMS*, *CuSPDS*, *CuACL5*, *CuADC*, *CuODC*, and *CuSAMDC* genes in the phylogenetic tree.

The results showed six main clades emerging from the rooted tree, forming six groups of polyamine biosynthetic genes: *SPMS*, *SPDS*, *ACL5*, *ADC*, *ODC*, and *SAMDC*. *SPMS* consists of five members with a single gene of *C. unshiu*, a single gene of *A. thaliana*, and three genes of *C. sinensis*; the *SPDS* group contains six members, two from each species; the *ACL5* group has four members, two of which are *C. unshiu*, with single genes of *A. thaliana* and *C. sinensis*; the *ADC* group consists of four members, two of which are *A. thaliana* genes, with one from *C. unshiu* and *C. sinensis*; the ODC group contains five members, three from *C. sinensis* and two from *C. unshiu*; and Arabidopsis is absent as it does not contain *ODC* in its genome. *SAMDC* is the largest group comprising thirteen members, with five from *C. unshiu*, while *A. thaliana* and *C. sinensis* has four members each (Figure 1).

It was noticed that *CuSPMS*, *CuSPDS*, *CuACL5*, *CuADC*, *CuODC*, and *CuSAMDC* genes appeared in the same clade in their respective groups, with *A. thaliana* and *C. sinensis*. Interestingly, *C. unshiu* and *C. sinensis* genes appeared to be in the same clade and subclades in most cases in all groups showing a very close relationship between both species (Figure 1).

Orthologous proteins in different species have been observed to show similar biological functions. In this study, *CuSPMS* showed up to 74% identity with *AtSPMS*, reportedly involved in growth, flowering, and defense response against bacteria. *CuSPDS1* and *CuSPDS2* had maximum percentage identity with *AtSPDS1* and *AtSPDS2*; these genes showed higher expression in pollens and flowers and are involved in inflorescence and overall plant growth. *CuACL5-1* and *CuACL5-2* were 75 and 61% identical to *AtACL5*, respectively, expressed in the whole plant, pollen, and flower and associated with apical leaf, inflorescent, and overall plant development. All these genes were localized both in the nucleus and cytoplasm. *CuADC* is identified as 61% identical to *AtADC1* expressed in the whole plant except the root and had been analyzed in Arabidopsis to have a role in shoot system, leaf, and inflorescence development. Arabidopsis lacks *ODC* genes, so the *CuODC* orthologs were analyzed in *C. sinenesis*.

*CuODC1* and *CuODC2* had maximum percentage identity of up to 100 and 92% with *CsODC1* and *CsODC2*, respectively, and expressed highly during the development to regulate leaf, inflorescence, shoot, root, and vascular system formation in citrus. *CuSAMDC1*, *CuSAMDC2*, *CuSAMDC3*, *CuSAMDC4*, *CuSAMDC5* were most identical to *AtSAMDC1*, *AtSAMDC2*, *AtSAMDC3*, and *AtSAMDC4*, respectively. These orthologs are expressed in the whole plant and highly at the mature and germinated pollen stage and have been found to significantly regulate guard cells, pollen, pollen tube, and organ development (Table S1).

### 3.3. Identification of Conserved Motifs and Gene Structural Analysis

**Figure 2:**
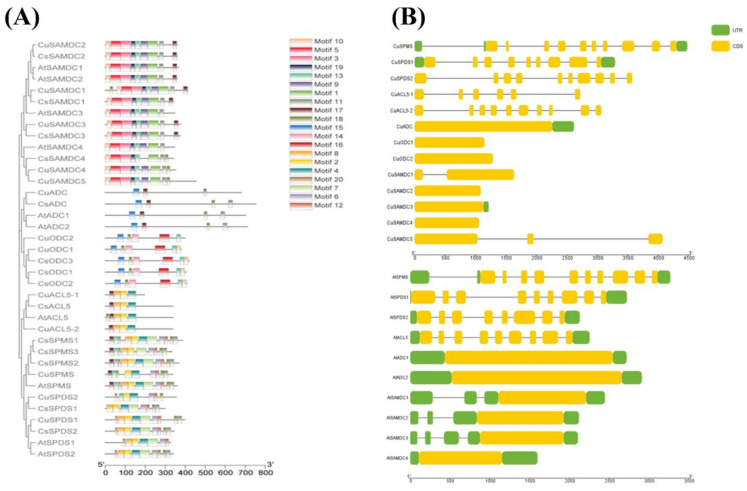
The distribution of conserved motifs and gene structure in PA biosynthetic proteins. (**A**) Identification of motifs using MEME version 5.4.1 and visual created by interlinking it with the phylogenetic tree through TBtools. The bars with color gradients represent various motifs with different color codes for each motif. (**B**) Gene structure and phylogeny of PA biosynthetic genes from *C. unshiu* and A. thaliana. Yellow bars indicate exons and black lines indicate introns in gene structure display of the *SPMS*, *SPDS*, *ACL5*, *ADC*, *ODC*, and *SAMDC* genes.

The MEME program studied the identification and distribution of 20 motifs within all the SPMS, SPDS, ACL5, ADC, ODC, and SAMDC proteins in C. unshiu, *C. sinensis*, and A. thaliana. Multiple motifs were also highly conserved among *SPMS*, *SPDS*, *ACL5*, *ADC*, *ODC*, and *SAMDC* proteins of *C. unshiu*, *A. thaliana*, and *C. sinensis* (Figure 2A).

The organization of exon and intron provide the backbones of genes and assist in verifying the study of the evolutionary relationships between genes or organisms. The number and distribution pattern of exon and intron are considered an evolutionary mark for a gene family. A comprehensive demonstration of the exon–intron structures of *C. unshiu* PA biosynthetic genes and their phylogeny analysis revealed that the pattern of gene structure was parallel to the phylogenetic analysis.

The *SPMS*, *SPDS*, *ACL5*, *ADC*, *ODC*, and *SAMDC* genes in the same clade showed similar gene structure patterns. Arabidopsis and *C. unshiu* displayed a somewhat similar number of introns and gene structures. Both *AtSPMS* and *CuSPMS* have ten exons, both *AtSPDS1* and *CuSPDS1* have nine exons, while *CuSPDS2* has ten and *AtSPDS2* has eight exons, *CuACL5-1* has six, *CuACL5-2* has ten, and *AtACL5* has nine exons. All other genes have just a single exon except *CuSAMDC1*, which has two, and *CuSAMDC5*, which has three exons (Figure 2B).

### 3.4. Cis-Elements Regulate the Transcriptional Activity of PA Biosynthetic Genes

**Figure 3:**
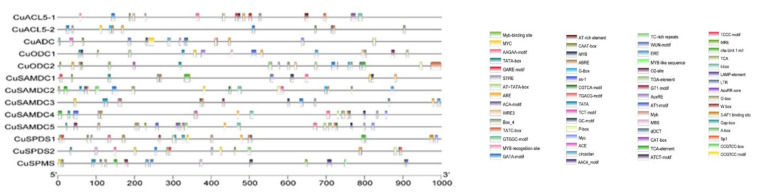
The presence and position of various cisacting elements on the promoter region of PA biosynthetic genes *C. unshiu*.

The presence and organization of various cisregulatory elements on the promoter region at the transcription factor binding site affect the genes’ spatial-temporal transcriptomic expression. Therefore, an in-silico analysis was conducted through the PlantCare database to evaluate the putative functions of genes. *Cis*acting elements were associated with hormone responses, light responses, defense and stress responses, and others involved in growth and development or acting as promoters and other binding sites found in the putative PA biosynthetic genes promoter region of *C. unshiu*. The position of the elements is represented on a scale of 1000 bps. Colored legend blocks at the bottom represent *cis*-elements found in each PA biosynthetic gene, with each element represented by a specific color (Figure 3 and Table S3).

*CuSPMS*, *CuSPDS*, *CuACL5*, *CuADC*, *CuODC*, and *CuSAMDC* genes of *C. unshiu* consisted of light-responsive and plant mechanisms-related, development-related, hormone-responsive, and stress- and defense-related cisregulatory elements. The *cis*regulatory elements identified among *CuSPMS*, *CuSPDS*, *CuACL5*, *CuADC*, *CuODC*, and *CuSAMDC* genes of *C. unshiu* and their functional annotation are shown in Figure 3. *CuSPMS*, *CuSPDS*, *CuACL5*, *CuADC*, *CuODC*, and *CuSAMDC* promoters incorporated 18 *cis*regulatory elements that are responsive to light and plant mechanisms, including 3-AF1 binding site, ACE, AT1-motif, ATCT-MOTIF, Box 4, chs-Unit 1 m1, Gap-box, GATA-motif, G-Box, GT1-motif, GTGGC-motif, I-box, LAMP-element, MRE, Sp1, TCCC-motif, and TCT-motif. Fourteen environmental stress-related elements as MYB, MYB-like, AAGAA-motif, ACA-moif, ARE, as-1, GC-motif, LTR, MBS, STRE, TC-rich repeats, W box, WRE3, and WUN-motif were also present. W box was only part of the *CuODC1* promoter and was absent in other genes. In addition, four development-related elements, including circadian, dOCT, MYC, and O2-site were also seen; MYC was found.

Seven promoter-related and site-binding *cis*elements like A-box, AT~TATA-box, AT-rich, CAAT-box, TATA-box, Myb-binding site, and MYB recognition site were also present in the genes; CAAT-box and TATA-box were found in abundance with as much as 73 in *CuODC1*; these are the most common transcription inducing *cis*-elements. However, MYB recognition site was present only in *CuSPMS*. Twelve hormone-responsive elements were also included in the promoter regions of PA biosynthetic genes involving an abscisic acid-responsive element ABRE, three auxin responsive elements AuxRE, AuxRR-core, and TGA-element, a methyl jasmonate-responsive CCGTCC motif, an ethylene-responsive ERE ciselements, three gibberellin responsive elements GARE, P-box, and TATC-box, and a salicylic acid-responsive TCA-element. Auxin-responsive *cis*element AuxRE and TGA were only present in *CuODC1* and did not appear in the promoter regions of any other gene.

### 3.5. Prediction Subcellular Localization Signals in PA Biosynthetic Genes of C. unshiu

Subcellular locations of *CuSPMS*, *CuSPDS*, *CuACL5*, *CuADC*, *CuODC*, and *CuSAMDC* genes were predicted in the cytoplasm, mitochondria, cytoskeleton, peroxisomes, plastid, etc., using WoLF PSORT (Figure S1). *CuSPMS* and *CuSPDS1* were highly localized in the cytoplasm. *CuSPDS2*, on the other hand, was almost equally located both in the cytoskeleton and nucleus; *CuACL5-1* was mostly located in the cytoplasm but also present in the nucleus in minute numbers, while *CuACL5-2* was present majorly in peroxisomes while a comparatively nonsignificant amount was located in the cytoplasm and the nucleus; *CuADC* was located mostly in both the chloroplast and the nucleus in almost equal amounts; *CuODC1* was mainly located in the cytoplasm, but it also occurred in cytoskeleton and vacuole, while *CuODC2* was only located in the chloroplast; *CuSAMDC1* was present in the nucleus but also in the cytoplasm and the chloroplast in a negligible amount; *CuSAMDC2*, *CuSAMDC3*, *CuSAMDC4*, and *CuSAMDC5* were distributed among the cytoplasm, the nucleus, the chloroplast, and the cytoskeleton.

### 3.6. Gene Location and synteny Analysis in C. unshiu PA Biosynthetic Genes

**Figure 4:**
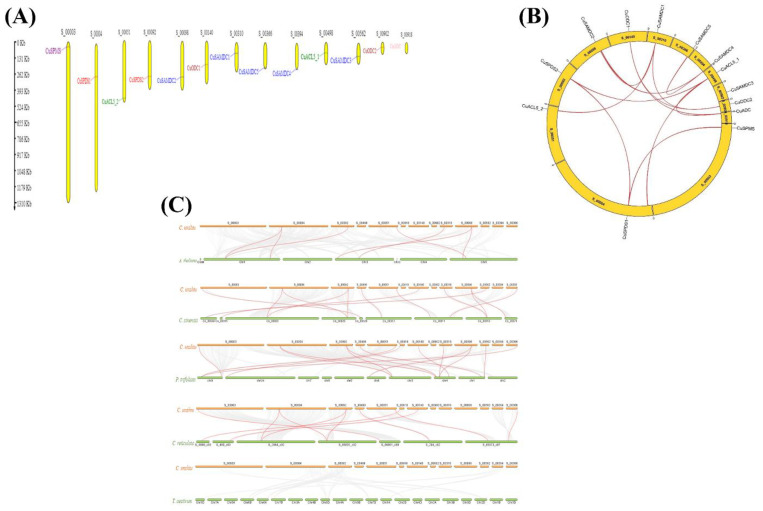
Gene location and synteny analysis. (**A**) Distribution of PA biosynthetic genes on scaffolds within *C. unshiu* genome. Yellow bars represent the scaffolds, and label on the top of each bar represents the scaffold number. (**B**) Syntenic relationship of PA biosynthetic genes within *C. unshiu* genome. Yellow curved blocks represent the scaffolds and the label on top of each scaffold in black color represents the scaffold number. Red lines indicate segmental or tandem duplication event gene pairs and rust-colored lines represent the position of PA biosynthetic genes on *C.unshiu* scaffolds. (**C**) Dual synteny of *C. unshiu* PA biosynthetic genes with various plant species. Colored horizontal bars represent chromosomes and label on top (*C. unshiu*) or bottom (other plant species) of these horizontal bars represent the chromosome number of the respective plant species.

Chromosomal distribution analysis of *C. unshiu* PA biosynthetic genes demonstrated that out of 13 scaffolds, *CuSPMS* was located on S_00003, *CuSPDS1* was located on S_00004, *CuSPDS2* on S_00092, *CuACL5-1* on S_00498, *CuACL5-2* on S_00051, *CuADC* on S_00918, *CuODC1* on S_00140, *CuODC2* on S_00902, *CuSAMDC1* on S_00310, *CuSAMDC2* on S_00098, *CuSAMDC3* on S_00562, *CuSAMDC4* on S_00394, and *CuSAMDC5* was located on S_00394 (Figure 4A).

Furthermore, syntenic analysis was performed for *CuSPMS*, *CuSPDS*, *CuACL5*, *CuADC*, *CuODC*, and *CuSAMDC* genes to gain insight into the probability of segmental or tandem duplication of the biosynthesis of the PA gene family in *C. unshiu*. *CuSPMS* had nine paralogs, *CuACL5-2* had four, *CuODC1* had ten, *CuSAMDC1* had eight, *CuSAMDC2* had seven, and *CuSAMDC5* had five paralogs. Whereas *CuSPDS1*, *CuSAMDC3*, *CuSAMDC4*, and *CuODC2* had six, and *CuSPDS2*, *CuADC* and *CuACL5-1* had nine paralogs each across different scaffolds across the *C. unshiu* genome (Figure 4B).

To understand the evolution of the biosynthesis of the PA gene family in *C. unshiu*, the syntenic relationship of *CuSPMS*, *CuSPDS*, *CuACL5*, *CuADC*, *CuODC*, and *CuSAMDC* genes with *A. thaliana*, *C. sinensis*, *P. trifoliata*, *C. reticulata*, and *Triticum aestivum* were analyzed. A number of *SPMS*, *SPDS*, *ACL5*, *ADC*, *ODC*, and *SAMDC* genes in *C. unshiu* were revealed as orthologous genes in *A. thaliana*, *C. sinensis*, *P. trifoliata*, *C. reticulata*, and *T. aestivum* through collinearity analysis (Figure 4C). All the PA biosynthetic genes in *C. unshiu* have a syntenic relationship with PA biosynthetic genes in all other species, i.e., *A. thaliana*, *C. sinensis*, *P. trifoliata*, and *C. reticulata*, except *T. aestivum* that had many genes linked with the *C. unshiu* genome but none of the *CuSPMS*, *CuSPDS*, *CuACL5*, *CuADC*, *CuODC*, and *CuSAMDC* members appeared to be linked with any gene of *T. aestivum*. In addition, *CuODC2* shows no links with any of the genes in other species. *CuSPMS* appeared to be linked with SPMS belonging to *C. sinensis*, *P. trifoliata*, and *C. reticulata*, but not with *A. thaliana*. *CuSPDS*, *CuACL5*, *CuADC*, *CuODC*, *and CuSAMDC* of *C. unshiu* showed syntenic relationships with gene members of their respective groups located on different chromosomes and scaffolds in *A. thaliana*, *C. sinensis*, *P. trifoliata*, and *C. reticulata*. The details of the linked genes of *CuSPMS*, *CuSPDS*, *CuACL5*, *CuADC*, *CuODC*, and *CuSAMDC* members of *C. unshiu* with other species, along with their locations, are described in Supplementary Table S2.

### 3.7. Evaluation of C. unshiu PA Biosynthetic Genes Duplication Event

**Figure 5:**
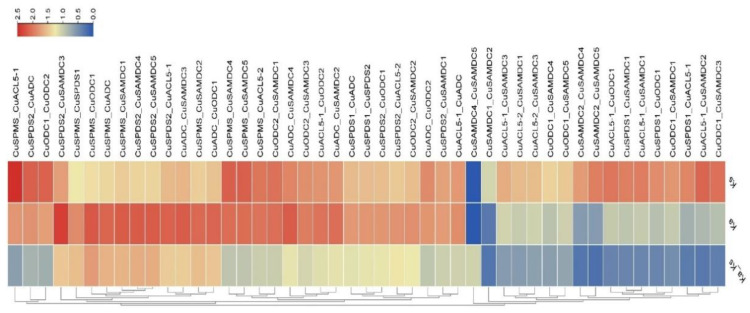
Ks and Ka values of PA biosynthetic gene pairs of *C. unshiu*. Color gradient represents values with blue representing the lowest and red representing the highest value.

The values of Ks, Ka, and Ka/Ks ratio of *CuSPMS*, *CuSPDS*, *CuACL5*, *CuADC*, *CuODC*, and *CuSAMDC* genes were estimated through TBtools using a simple Ka/Ks calculator (Figure 5). Forty-seven gene pairs of *CuSPMS*, *CuSPDS*, *CuACL5*, *CuADC*, *CuODC*, and *CuSAMDC* members appeared and all the genes function are listed in Table S4. Ks of a gene pair depicts the number of synonymous substitutions per synonymous site. In contrast, Ka shows the number of nonsynonymous substitutions per nonsynonymous site, and the ratio of nonsynonymous (Ka) versus synonymous (Ks) mutation was represented by Ka/Ks. This ratio ranged from 0.174 in the *CuSAMDC2_CuSAMDC5* pair to 2.412 in the *CuSPMS_CuODC1* pair. Up to 22 paralogous group pairs in *C. unshiu* had Ka/Ks ratio of less than 1, which suggested the probability of limited functional divergence in the duplication process due to the purifying selection among these paralogs.

### 3.8. Expression of C. unshiu PA Biosynthetic Genes during Peel Roughing Disease

**Figure 6:**
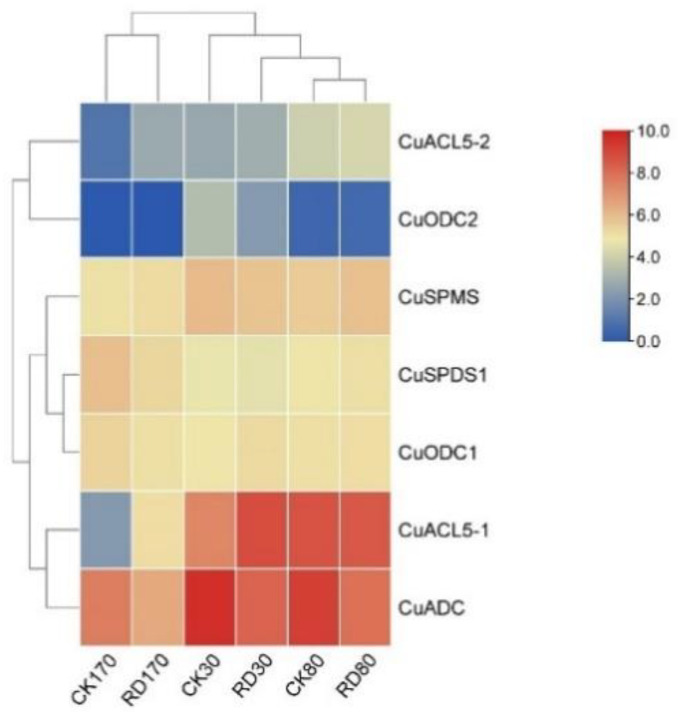
Heat map showing expression of *C. unshiu* PA biosynthetic genes exposed to peel roughing disease. The color gradient represents the relative expression of these genes, with red indicating the highest and blue indicating the lowest level of expression.

The results demonstrated that *CuADC* showed the highest expression in control after 30 and 80 days and continued to drop significantly in plants from day 30 to 170 (Figure 6). After exposure to the rough disease of mandarin, expression of *CuADC* declined drastically, and a little more than a two-fold difference was observed between the control and affected plant. However, *CuACL-5* expression increased from day 30 to day 80 more than twice in the control group. Interestingly, the expression of the *CuACL-5* gene was highest in the affected group, even more than *CuADC*. A sudden surge in *CuACL-5* expression was noticed after 30 days, the difference was almost three-fold between the affected and control group, but it continued to decline afterwards. Overall, the expression of all the PA biosynthetic genes of *C. unshiu* was the lowest after 170 days in both control and affected groups.

### 3.9. C. unshiu PA Biosynthetic Genes Showing Fluctuation in Expression during Seedling Etiolation in Two Citrus Hybrids

**Figure 7:**
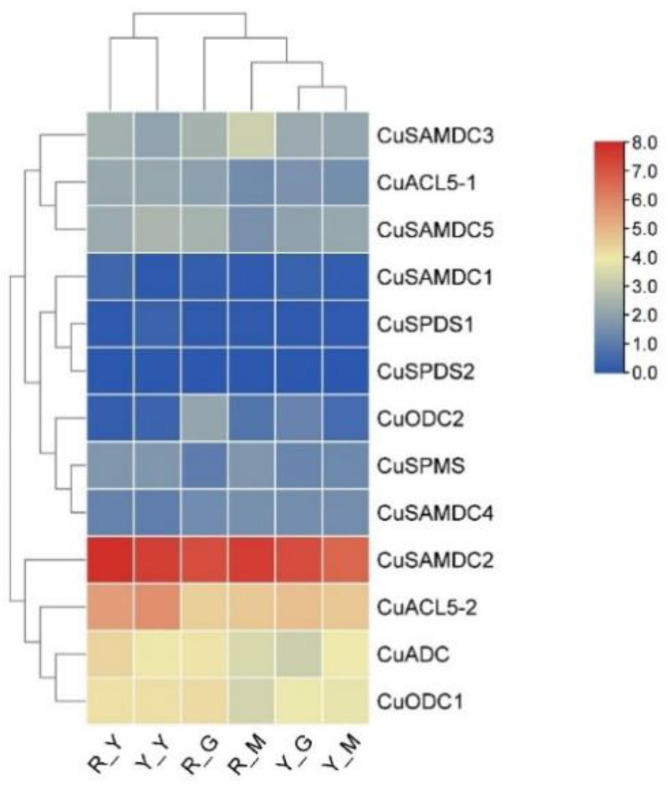
Heat map showing *C. unshiu* PA biosynthetic gene expression in seedlings during etiolation. The seedling groups as R_G, Shiranuhi green seedlings; R_Y, Shiranuhi etiolated seedlings; R_M, Shiranuhi multicolored seedlings; Y_G, Huangguogan green seedlings; Y_Y, Huangguogan etiolated seedlings; and Y_M, Huangguogan multicolored seedlings. The color gradient represents the relative expression of these genes, with red indicating the highest and blue indicating the lowest level of expression.

The transcriptomic data showed the expression of all *C. unshiu* PA biosynthetic genes, i.e., *CuSPMS*, *CuSPDS*, *CuACL5*, *CuADC*, *CuODC*, and *CuSAMDC*, during the etiolation of both Shiranuhi and Huangguogan seedlings; however, only the expression showed by *CuACL5-1*, *CuADC*, *CuODC2*, *CuSAMDC2*, *CuSAMDC3* and *CuSAMDC5* was statistically significant (*p*-value ≤ 0.05) (Figure 7). In Shiranuhi, *CuACL5-1*, *CuADC*, and *CuSAMDC2* showed significant upregulation in etiolated seedlings, and *CuODC2* was significantly upregulated in green seedlings, while *CuSAMDC3* was upregulated significantly in multicolored seedlings (Figure 7). However, in Huangguogan only, *CuSAMDC5* showed significant upregulation in etiolated seedlings. The remaining genes were mostly downregulated, or their expression remained unchanged in Shiranuhi and Huangguogan seedlings.

### 3.10. Differential Expression of PA Biosynthetic Genes in the Development of Light Drought-Induced Flowering Branches in C. unshiu

**Figure 8:**
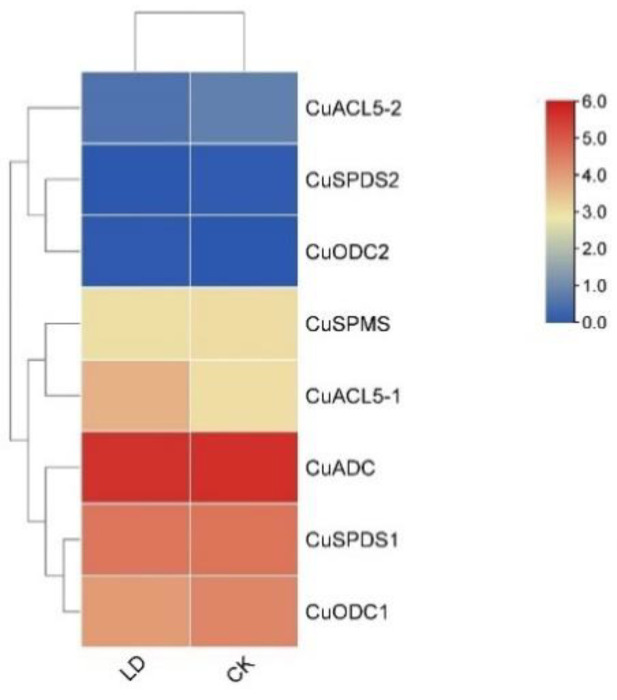
Gene expression of PA biosynthetic genes under light drought (LD) stress in *C. unshiu* represented through a heatmap. The color gradient varies from blue, indicating the lowest expression level, to red, indicating the highest expression level of PA biosynthetic genes.

In this study, *CuSPMS*, *CuSPDS1*, *CuSPDS2*, *CuACL5-1*, *CuACL5-2*, *CuADC*, *CuODC1*, and *CuODC2* appeared to be expressed in flowering branches after 75 days in those exposed to light drought (LD) compared with a conventionally watered group (CK). However, the heatmap revealed that the expressions of *CuSPDS2*, *CuACL5-2*, and *CuADC2* were nonsignificant (Figure 8). *CuADC* expression was the highest among all the expressed PA biosynthetic genes in both LD and CK groups. The expression of *CuSPDS1* and *CuACL5-1* was about the same in both CK and LD groups. A raise in the expression of *CuADC* and *CuODC1* was observed in LD; on the other hand, *CuACL5-1* expression dropped in the LD group when compared with the control group.

## 4. Discussion

In this study, the *C. unshiu* was used to identify the biosynthesis of the PA gene family at the genome level in satsuma mandarin (*C. unshiu*). A total of single *SPMS*, two *SPDS*, two *ACL5*, single *ADC*, two *ODC*, and five *SAMDC* nonredundant PA biosynthetic genes were identified in *C. unshiu*. The prediction of subcellular localization signals confirmed the abundant location of *CuSPMS*, *CuSPDS*, *CuACL5*, *CuADC*, *CuODC*, and *CuSAMDC* genes that were predicted in the cytoplasm, mitochondria, cytoskeleton, peroxisomes, and plastid, etc.

Knowledge about the presence and position of exons and introns in a gene can be used to understand the gene’s evolutionary relationship with other genes or organisms [40,41]. Generally, a similar exon–intron structure was shared among genes, and different groups showed different structures. A similar structure of exon and intron was noticed in Arabidopsis. The results suggest that these structures remain preserved during the evolutionary process and might play a role in protecting gene integrity.

Phylogenetic analysis of PA biosynthetic genes was conducted in *C. unshiu*, *A. thaliana*, and *C. sinensis*. Based on their domains, all PA biosynthetic genes were classified into six subgroups of polyamine biosynthetic genes: *SPMS*, *SPDS*, *ACL5*, *ADC*, *ODC*, and *SAMDC*. Arabidopsis PA biosynthetic genes showed a close phylogenetic relationship with PA biosynthetic genes in *C. unshiu*, as they appeared to be in the same subclades. PA biosynthetic genes in Arabidopsis were mostly localized in the cytoplasm, nucleus, and chloroplast [42], and were expressed in the whole plant except *AtADC1*, and *AtSAMDC* members were not expressed in roots, and significantly expressed in embryo development, inflorescence, and pollen. *AtSPMS* was also involved in defense response to bacteria. However, *CuODC1* and *CuODC2* were highly homologous to *CsODC1* and *CsODC2*, respectively. Both these genes are important in the shoot system and vascular system development in *C. sinensis*, in addition to leaf and inflorescence development. *AtSAMDC* genes were only localized in the cytosol and had a role in the guard cells, pollen tube, and organ development [43]. Due to the high protein homology of *C. unshiu* and Arabidopsis and *C. sinensis* genes, it can be hypothesized that PA biosynthetic genes might perform in the same pattern in *C. unshiu*.

The linkage of genes in the genome of the same species can assist in predicting the gene duplication event. The linked genes have a chance that they might result from segmental duplication of a similar gene or whole genome duplication (WGD) [44]. PA biosynthetic genes appeared to have multiple paralogs, most importantly with other PA biosynthetic genes across the *C. unshiu* genome. Many genes appeared to be interlinked with multiple PA biosynthetic genes, which indicates that these genes may have evolved together and may result from segmental duplication of a similar type of gene. There is a possibility that the increase in PA biosynthetic genes might be due to the duplication of their *SPMS*, *SPDS*, *ACL5*, *ADC*, *ODC*, and *SAMDC* domains during the evolution of eukaryotic plants [45,46].

Comparative syntenic maps can be used further to explore the potential evolutionary mechanism of a gene family [47]. Syntenic maps of *C. unshiu* were constructed comparatively to the genomes of *A. thaliana*, *C. sinensis*, *P. trifoliata*, *C. reticulata*, and *T. aestivum* to better understand. Many genes from these species were linked with the *C. unshiu* genome, but none of the *CuSPMS*, *CuSPDS*, *CuACL5*, *CuADC*, *CuODC*, and *CuSAMDC* members appeared to be linked with any gene of *T. aestivum*. This may happen due to a higher degree of variation from monocots to dicots during evolution. However, in *Arabidopsis*, citrus species, and trifoliate orange *CuSPMS*, *CuSPDS*, *CuACL5*, *CuADC*, *CuODC*, and *CuSAMDC* members appeared to have syntenic pairs with genes of respective groups, indicating that duplicated genes were conserved among these species.

The selection pressure on substituting amino acids can be understood through the ratio of Ka/Ks. Ka/Ks < 1 ratio suggests the possibility of a purifying selection, whereas Ka/Ks ratio > 1 suggests the likelihood of a positive selection [48,49]. Generally, evaluating selective pressure provides a selective lead for amino acid sequence altered in a protein and is also necessary for interpreting functional residues and protein shifts [50]. Forty-seven gene pairs appeared among *CuSPMS*, *CuSPDS*, *CuACL5*, *CuADC*, *CuODC*, and *CuSAMDC* members, and twenty-two paralogous pairs had Ka/Ks ratio of less than 1, indicating that these genes went through strong purifying selection pressure and a positive selection might have acted on a few sites only during the process of evolution among these paralogs.

The occurrence of hormone-responsive, stress and defense-responsive cisacting elements indicates that these genes might have important roles in plant growth and development and might be involved in plant defense mechanisms in *C. unshiu*. Eighteen light-responsive and plant mechanisms-related cisregulatory elements were found in the promoter regions of *CuSPMS*, *CuSPDS*, *CuACL5*, *CuADC*, *CuODC*, and *CuSAMDC*; the number varied for a few members, which indicates their role in photosynthesis and leaf development related roles like their orthologs. The abundance of promoter-related ciselements such as 73 TATA-box in *CuODC1* indicates their expression during normal growth conditions.

PA biosynthetic genes of *C. unshiu* showed expression in various organs and developmental stages under stressed and normal conditions. As described earlier, various PA biosynthetic genes of *C. unshiu* were found to be expressed in the profiling data downloaded from NCBI GEO experimental datasets [51,52]. Using expression data to categorize differentially expressed genes (DEGs) is a very efficient way to investigate the gene interaction, signaling, and functions of targeted genes [53]. Genes with similar expression patterns usually have functional analogies [54]. After exposure to the rough disease of mandarin, expression of *CuADC* declined drastically, and a little more than a two-fold difference was seen among the affected and control plants than control groups. However, a sudden surge in *CuACL-5* expression was noticed after 30 days, the difference was almost three-fold between the affected and control group, but it continued to decline afterwards. The results indicate that the peel deformation may be related to decreased *CuADC* and *CuACL-5* activity. The rise in *CuACL-5* expression after 30 days may respond to the disease initiation, affecting cell differentiation in the peel.

## 5. Conclusions

A detailed analysis of the biosynthesis of the polyamine gene family in satsuma mandarin (*C. unshiu*) was reported in this study. Thirteen PA biosynthetic genes were identified and categorized into six subgroups (*SPMS*, *SPDS*, *ACL5*, *ADC*, *ODC*, and *SAMDC*). Domain motif and intron–exon analysis revealed that the basic gene structure remained conserved throughout the evolutionary process. The biosynthesis of the PA gene family members in the genome of various plant species, such as seven members in Arabidopsis, and thirteen in *C. sinensis*, suggests that these genes might have emerged because of either whole-genome (segmental) duplication or a tandem duplication event, which ultimately led to the expansion of the biosynthesis of the PA gene family in various plant species. PA biosynthetic genes showed a strong interaction with other members of glutathione, cysteine, methionine, arginine, and proline metabolism, and interruption in their gene expression might severely disrupt these metabolic pathways in *C. unshiu*. The involvement of PA biosynthetic genes, mostly in abiotic and biotic stress tolerance mechanisms and during developmental stages, was analyzed. A high expression of various PA biosynthetic genes during light, drought, flower induction, roughing disease, and the presence of stress; light, hormone-responsive, and plant mechanism-related ciselements highlight the significance during stress and development. Detailed in-silico analysis of the biosynthesis of the PA gene family in *C. unshiu* revealed in the current study might assist in understanding the mechanism of the polyamine biosynthesis pathway and regulation during various developmental stages and in response to different stresses in *C. unshiu* at the molecular level.

## Figures and Tables

**Table 1:** List of identified PA biosynthetic genes in *C. unshiu* genome.

Gene ID	Gene Name	Scaffold ID	Start	End	D	AA	MW (Kda)	pI
C_unshiu_00003:mRNA_4.1	*CuSPMS*	C_unshiu_00003	40193	44670	R	338	38653.7	5.5
C_unshiu_00004:mRNA_33.1	*CuSPDS1*	C_unshiu_00004	260305	263596	R	399	44011.5	5.2
C_unshiu_00092:mRNA_48.1	*CuSPDS2*	C_unshiu_00092	340652	344223	R	356	40805.8	5.3
C_unshiu_00498:mRNA_14.1	*CuACL5-1*	C_unshiu_00498	87851	90568	R	196	22151.4	5.6
C_unshiu_00051:mRNA_69.1	*CuACL5-2*	C_unshiu_00051	560073	563136	R	337	37747.7	5.4
C_unshiu_00918:mRNA_1.1	*CuADC*	C_unshiu_00918	5010	7626	F	681	76854.2	5.1
C_unshiu_00140:mRNA_18.1	*CuODC1*	C_unshiu_00140	217799	218944	F	381	41218	5.7
C_unshiu_00902:mRNA_4.1	*CuODC2*	C_unshiu_00902	47979	49265	F	399	45316.1	7.2
C_unshiu_00310:mRNA_10.1	*CuSAMDC1*	C_unshiu_00310	93929	95562	F	413	45414.5	5.2
C_unshiu_00098:mRNA_50.1	*CuSAMDC2*	C_unshiu_00098	332348	333433	F	361	39849.1	5
C_unshiu_00562:mRNA_14.1	*CuSAMDC3*	C_unshiu_00562	106938	108153	R	376	40852.4	5.5
C_unshiu_00394:mRNA_35.1	*CuSAMDC4*	C_unshiu_00394	222794	223852	F	352	39289.6	5.2
C_unshiu_00366:mRNA_33.1	*CuSAMDC5*	C_unshiu_00366	221007	225078	F	456	51360.6	5.6

(Note: D = Direction, R = Reverse, F = Forward, AA = Amino Acid, MW = Molecular Weight, pI = isoelectric point).

## Data Availability

Not applicable.

## Supplementary Materials

The following supporting information can be downloaded at: https://www.mdpi.com/article/10.3390/genes14081527/s1, Table S1: Brief information about *C. unshiu* SPMS, SPDS, ACL5, ADC, ODC, and SAMDC genes orthologs and their functions and expression in Arabidopsis. Table S2: Details of the linked genes of CuSPMS, CuSPDS, CuACL5, CuADC, CuODC, and Cu-SAMDC members of *C. unshiu* with other species, along with their locations. Table S3: Brief information about *C. unshiu SPMS, SPDS, ACL5, ADC, ODC*, and *SAMDC* genes orthologs and their functions and expression in Arabidopsis. Table S4: Brief information about *C. unshiu* SPMS, SPDS, ACL5, ADC, ODC, and SAMDC genes orthologs and their functions via molecular, interactiona and pathway. Figure S1: Heatmap illustration and distribution of sub-cellular localization.
