# Supplementary figures and images for: Genome-Wide Identification and Characterization of the Biosynthesis of the Polyamine Gene Family in Citrus unshiu
Source: Genes (Basel). 2023 Jul 26;14(8):1527. doi: 10.3390/genes14081527 (PMC10454681; doi:10.3390/genes14081527)

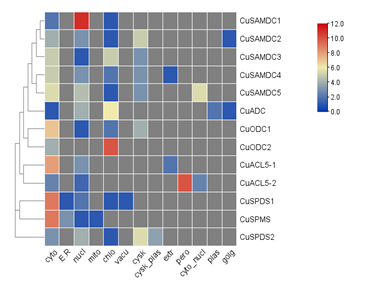

Supplement: Supplementary file 1 [file genes-14-01527-s001.zip › Figure S1.png]
